# Renal cortical hypoperfusion caused by glyphosate–surfactant herbicide

**DOI:** 10.1007/s10157-019-01691-z

**Published:** 2019-01-19

**Authors:** Takashin Nakayama, Yu Mimura, Keita Hirano

**Affiliations:** 1 Department of Internal Medicine, Ashikaga Red Cross Hospital, Tochigi, 284-1 Yobe, Ashikaga, Tochigi 326-0843 Japan; 2 Department of Psychiatry, Ashikaga Red Cross Hospital, Tochigi, 284-1 Yobe, Ashikaga, Tochigi 326-0843 Japan

**Keywords:** Renal cortical necrosis, Reverse rim sign, Glyphosate–surfactant herbicide

**Fig. 1:**
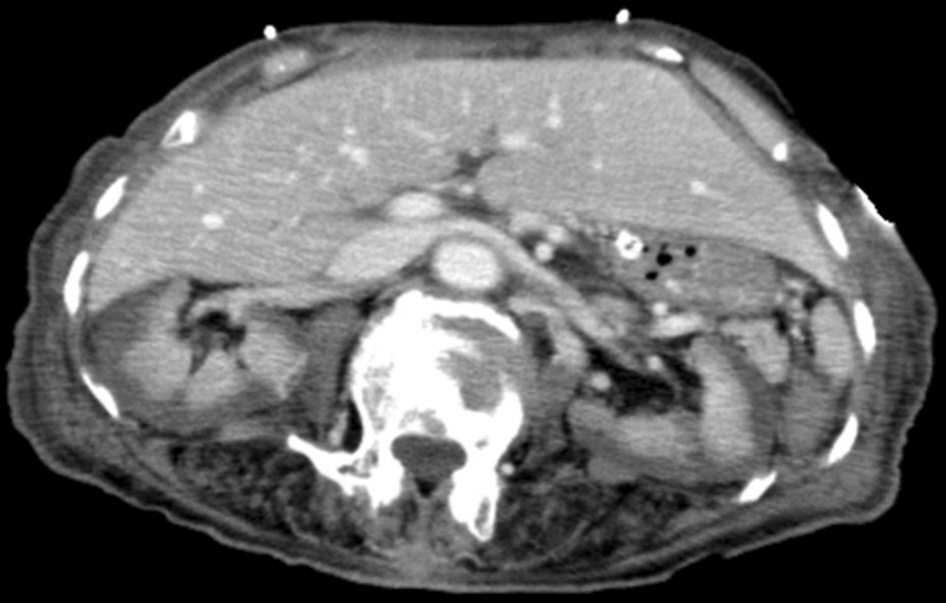
Contrast-enhanced computed tomography in the axial view demonstrating a non-enhancing renal cortex against a background of intact medullary perfusion

A 70-year-old woman presented with abdominal pain 2 h after ingesting 500 ml of glyphosate–surfactant herbicide (GPSH). As her abdominal pain worsened, contrast-enhanced computed tomography was performed 12 h after the admission, demonstrating renal blood flow shunting through the medulla which is called “reverse rim sign” (Fig. 1). She had been anuric since hospitalization with sufficient mean artery pressure and negative blood culture. Despite intensive care, she died of multiple organ failure on the 6th day of hospitalization.

Following ingestion of GPSH known for mitochondrial toxicity, our patient developed acute kidney injury (AKI) with diffuse renal cortical hypoperfusion [1]. We assumed that her AKI was attributed to vasoconstriction of small intracortical blood vessels, intravascular thrombosis or mitochondrial toxicity of GSPH resulting in disorders of the mitochondrion-rich renal cortical region [2]. Considering that the lesion was homogeneously localized to the renal cortex, the latter assumption seems to be unignorable.
